# Use of yoga in outpatient eating disorder treatment: a pilot study

**DOI:** 10.1186/s40337-016-0130-2

**Published:** 2016-12-09

**Authors:** Allison Hall, Nana Ama Ofei-Tenkorang, Jason T. Machan, Catherine M. Gordon

**Affiliations:** 1 Division of Adolescent Medicine, Hasbro Children’s Hospital, Providence, RI USA; 2 Division of Adolescent and Transition Medicine, Cincinnati Children’s Hospital Medical Center, 3333 Burnet Ave, MLC 4000, Cincinnati, OH 45229 USA; 3 Department of Orthopaedics & Surgery, Brown University, Providence, RI USA; 4 Biostatistics Core, Rhode Island Hospital, Providence, RI USA

**Keywords:** Eating disorders, Adolescents, Yoga, Anxiety, Depression, Body image

## Abstract

**Background:**

Individuals with restrictive eating disorders present with co-morbid psychiatric disorders and many attempt to control symptoms using strenuous exercises that increase caloric expenditure. Yoga offers a safe avenue for the engagement in physical activity while providing an outlet for disease-associated symptoms. This study sought to examine use of yoga practice in an outpatient setting and its impact on anxiety, depression and body image disturbance in adolescents with eating disorders.

**Methods:**

Twenty adolescent girls were recruited from an urban eating disorders clinic who participated in weekly yoga classes at a local studio, in addition to standard multidisciplinary care. Yoga instructors underwent training regarding this patient population. Participants completed questionnaires focused on anxiety, depression and body image disturbance prior to the first class, and following completion of 6 and 12 classes.

**Results:**

In participants who completed the study, a statistically significant decrease in anxiety, depression, and body image disturbance was seen, including: Spielberger State anxiety mean scores decreased after the completion of 7–12 yoga classes [47 (95%CI 42–52) to 42 (95%CI 37–47), adj. *p* = 0.0316]; as did the anorexia nervosa scale [10 (95% CI 7–12) vs. 6 (95%CI 4–8), adj. *p* = .0004], scores on Beck depression scales [18 (95%CI 15–22) to 10 (95%CI 6–14), adj. *p* = .0001], and weight and shape concern scores [16 (95%CI 12–20) to 12 (95%CI 8–16), adj. *p* =0.0120] and [31 (95%CI 25–37) to 20 (95%CI 13–27), adj. *p* = 0.0034], respectively. No significant changes in body mass index were seen throughout the trial.

**Conclusions:**

Yoga practice combined with outpatient eating disorder treatment were shown to decrease anxiety, depression, and body image disturbance without negatively impacting weight. These preliminary results suggest yoga to be a promising adjunct treatment strategy, along with standard multidisciplinary care. However, whether yoga should be endorsed as a standard component of outpatient eating disorder treatment merits further study.

## Plain English summary

Twenty adolescent girls with eating disorders, age 14–18 years, were recruited from an urban clinic to participate in a 12-week yoga intervention. The purpose of this study was to learn whether yoga can reduce depression and anxiety, improve overall mood, and promote self-acceptance in this patient population. Participants were required to attend one yoga class of their choice weekly at a selected local studio and to complete psychological questionnaires at 6 and 12 weeks that assessed changes in anxiety, depression and mood. Using psychological assessment tools, decreases in depression, anxiety, and eating disordered thoughts were seen, suggesting that yoga practice may augment standard multidisciplinary care (which provides medical, nutritional, and psychological support). These results suggest yoga to be safe for these patients and may be a novel and important addition to their care. However, larger, more definitive studies are needed before yoga can be endorsed as a component of standard care for adolescents with eating disorders.

## Background

Eating disorders are complicated medical and psychological conditions that are increasing in prevalence, affecting 1–5% of adolescent girls in the United States [1]. Affected individuals often present with co-morbid psychiatric disorders including depressive symptoms and anxiety disorders [2, 3]. Individuals with these disorders frequently attempt to control psychological symptoms through hyperexercise [4]. Therefore, most clinicians prohibit exercise and inform patients that engagement in strenuous activity may increase caloric expenditure and worsen prognosis [5]. Yoga has been shown previously to be equal or superior to other aerobic activities in improving a number of outcomes such as attention, alertness and energy expenditure, associated with chronic conditions (e.g., chronic renal failure, multiple sclerosis, schizophrenia, etc.) [6].

Yoga is an intervention that addresses movement, breathing, and awareness of bodily sensations; it helps to increase awareness of internal states and reorganize physiological responses connected to symptoms. Originating in India, it is a practice designed to create a sense of well-being, improve self-confidence and efficiency, increase attentiveness, and provide an optimistic outlook [7]. Comprised of physical and mental disciplines, yoga improves the body’s sense of embodiment and interoception. A lack of interoceptive awareness is a key characteristic of anorexia nervosa and bulimia nervosa [8]. One aspect of the eight-limbed yoga philosophy involves specific postures called *asanas*, through which attention is focused inward and the practitioner transcends the mind-body divide in an attempt to experience the true self or soul [9].

There has been increasing interest regarding the therapeutic benefits of yoga to prevent or treat many medical conditions, as well as mental health disorders [10]. Psychological and physical benefits that may be derived from the practice of such mind-body activities has been studied in healthy high school students [11, 12]. Preliminary work suggests yoga to be of benefit for anxiety, depression and general eating disorder symptoms [13]. Studies exploring yoga in the eating disorder population have been few and carried out primarily in adults, but have shown improvement in depression and anxiety [14, 15]. Other studies in adolescents have included the effect of yoga on quality of life, and health outcomes and adherence in teenagers with hemophilia and asthma [16, 17]. The only randomized controlled trial to date that has assessed the impact of yoga on eating disorder outcomes found that yoga treatment decreased weight and shape concerns [18].

Given that yoga offers a framework (including gentle restorative poses, breath work, and meditation) for helping individuals to connect with their bodies in a healthy way, these classes may provide a venue for improvement of eating disordered symptoms, and its associated anxiety and depression. Yoga involves low aerobic intensity and affords an opportunity for physical activity [19, 20], while avoiding weight loss and excessive caloric expenditure. In this pilot study, we tested the adherence of adolescent girls with eating disorders to a 12-week protocol involving once weekly yoga classes. We also obtained preliminary data on yoga as an adjunct to multidisciplinary outpatient care for adolescents with eating disorders, as a strategy to reduce stress and anxiety and promote self-acceptance. We explored whether participants would present with lower levels of anxiety, depression, and body image concerns after engagement in yoga. We introduced yoga to potential study participants as a non-threatening means for improvement of eating disordered symptoms and associated anxiety and depression, while helping to strengthen the mind-body connection.

## Methods

### Study design and recruitment methods

This outpatient yoga pilot program, entitled “Let’s Yoga,” enrolled female patients, age 11–18 years, who met DSM-5 criteria for anorexia nervosa, bulimia nervosa, avoidant restrictive food intake disorder, or other specified feeding or eating disorder. All study recruits were active participants in the same outpatient clinic where they received medical monitoring, nutritional counseling and social work intervention. The clinic followed guidelines established by the Society of Adolescent Health and Medicine regarding management of eating disorders [1].

Patient recruitment was achieved through a brief conversation at a scheduled clinic visit or phone response to a flyer sent via mail to all patients who met the age criteria. We excluded patients who presented with severe depression (e.g., voicing harm to themselves or actively suicidal) and patients who were not hemodynamically stable (e.g., low pulse or blood pressure). Patients who were currently hospitalized or had other chronic medical conditions in addition to the eating disorder (e.g., cystic fibrosis, sickle cell anemia) were also excluded.

After expressing interest in the study, a social worker or study coordinator reviewed study details with the patient. Once interest was confirmed, the primary eating disorders medical provider was contacted to obtain medical clearance for participation. Stability was determined based on the patient’s adherence to treatment, body mass index (BMI kg/m^2^) and vital signs.

The research coordinator scheduled the first study visit to obtain baseline measures. Blood pressure, heart rate, and respiratory rate were recorded. Height and weight data, blinded from the patient, were obtained using a single calibrated scale (in a gown) and stadiometer. A BMI was calculated using the calculator on the Centers for Disease Control and Prevention website. After completion of the first visit, each participant received a yoga mat, punch card to track class attendance, and a free pass for parent participation in a yoga class. All study visits took place in the hospital’s clinical research center.

### Yoga intervention

Following the baseline visit, participants attended a yoga class once weekly, at the identified local yoga studio. Class choice was limited to non-heated hatha-based classes identified by the studio owner as gentle in practice. Yoga techniques and class aims used in the study are presented in Table 1. The yoga class ranged between 60 and 90 min, Monday through Sunday. Instructors were comprised of certified yoga instructors trained at the minimum 200-h level certification through the Yoga Alliance, some of whom were also licensed clinical social workers or certified school teachers. Most instructors had more than 1000 h on the mat experience and many had specialty training in trauma sensitive curriculum. Instructors also underwent a training session provided by the research social worker regarding working with the study population.

**Table 1:** Yoga class techniques and aims

Techniques
Inversions
Arm balances
Deep breathing
Sanskrit chanting
Seated meditation
Sun salutations + flow
Restoration postures
Postures + movement
Postures + rhythmic breathing
Music: pop, hip hop, and rock music
Aims
Relaxation
Strengthen body
Increase flexibility
Release tension in the mind and body
Strengthening and lengthening the body
Release emotional stresses stored in the body
Target connective tissues, ligaments, bones, and body joints
Constant mindfulness: how is the body feeling in this moment?
Integration mind, body, and soul to reduce anxiety, depression or stress

### Follow up visits assessments

Follow up visits occurred after the completion of 6 and 12 classes, amounting to two visits after the baseline evaluation. Vital signs were recorded at each visit, height and weight measures (blinded from the patient) were obtained, and a BMI calculated.

### Psychological assessments

At all study visits, participants provided self-reported ratings on individual health items including physical activity, smoking and alcohol consumption status. Completion of validated psychological questionnaires were used to evaluate mood changes prior to and during the intervention: State of Mind Questionnaire, Spielberger State-Trait Anxiety Inventory, Eating Attitudes Test 26-Item (EAT-26), and the Eating Disorder Examination Questionnaire (EDE-Q).

*State of Mind Questionnaire (SOM)* is a combination of the Beck Depression Inventory (BDI) and the “Anorexia” scale. The BDI is a 21-item questionnaire used to index symptoms of severe depression. Using a four-point scale which ranges from 0 (symptoms not present) to 3 (symptoms very intense), the first portion assesses psychological symptoms such as irritability and feelings of being punished while the second assesses physical symptoms such as weight loss and fatigue. The Anorexia scale measures change of clinical state in anorexia nervosa by assessing body image distortions and feelings of inadequacy which lead to depression [21–23].

*State Trait Anxiety Inventory* consists of 40 items divided into two scales: state and trait anxiety. State anxiety refers to the short-lived emotion characterized by physiological arousal and consciously perceived feeling of tension. Trait anxiety refers to a person’s overall anxiety. All items are rated on a four point Likert scale and adolescent normative samples have shown good test-retest correlations and confirm validity [24–26].

*Eating Attitudes Test (EAT)* is used to measure symptoms and concerns characterized of eating disorders. It has been a successful screening tool to assess eating disorder risk in adolescents. A score of 20 or higher indicates the likelihood of a serious eating disorder [27–29].

*Eating Disorder Examination-Questionnaire (EDE-Q 6.0)* is a self-report questionnaire version of the Eating Disorder Examination interview which has been an acceptable indication of overall eating pathology based on reliability, validity, and normative data. It is comprised of four clinically derived subscales: restraint, eating concern, weight concern and shape concern, consisting of five-eight items each. Each item is rated using a seven-point forced-choice scale (0–6) with higher scores representing greater pathology [30–32].

### Participant incentives

To maintain and provide the assurance of anonymity among study participants, punch cards given to each study subject matched those used by others (non-research participants) who attended the same studio. At study completion, participants were given a cash incentive and gas cards to cover travel costs. The cost of all yoga classes was covered and a yoga mat provided.

### Statistical analysis

SAS version 9.4 (The SAS Institute, Cary, NC) was used to conduct statistical analyses. Generalized estimating equations were used to test for changes over time, by treating within participant responses as having correlated error. Analysis followed the intention to treat principle with data from all participants who started the study included in the final analysis, with baseline scores carried over for those who discontinued the protocol. The distribution chosen for most dependent variables was Gaussian after review of model residuals, reducing these models to equivalent repeated measures analysis of variance. A binomial based model was used for the Beck Depression Inventory as participant scores were approaching the lower limit of the scale. In all models, classical sandwich estimation was used to adjust for model misspecification and alpha was maintained at 0.05 across the three pairwise comparisons between time points using the Holm test. Cross-sectional means and 95% confidence intervals are presented, but it should be noted that for variability within-participants, changes were reduced based on the degree of within-participant correlation. Figures were produced showing individual participant scores, as well as means with 95% confidence intervals.

## Results

**Fig. 1:**
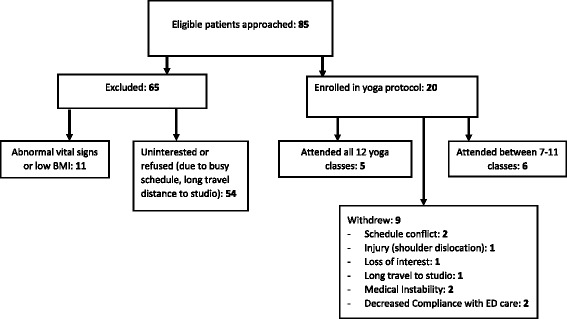
Consort diagram depicting study recruitment, enrollment and attrition

A total of 85 patients were approached for potential study participation (Fig. 1); 20 were enrolled. Physical and demographic characteristics, including age, weight-height ratios and BMI, and diagnostic information are presented in Table 2. Of the 65 who did not participate, 11 (17%) were excluded reporting low BMI and/or abnormal vital signs. The remaining 54 (83%) were uninterested or refused due to busy schedules and longer travel distance to the local studio. A total of nine participants discontinued the study and reasons for attrition included schedule conflicts, injury due to other cause (i.e., shoulder dislocation), loss of interest, medical instability, and decreased compliance with scheduled eating disorder clinic visits. The average length to complete the study was 20 weeks (range 12–29 weeks), and vital signs and BMI obtained at all visits remained stable throughout participation. Because of unexpected challenges encountered in subjects adhering to the weekly class regimen, we obtained assessments and report data after completion of the 6th and then 12th classes, and noted the associated duration of time. The time required to complete the 12 classes was longer than expected. The time range was 6 to 19 weeks from the baseline to 6th class assessment, as well as the 6^th^ to 12^th^ class assessment.

**Table 2:** Participant demographics

Measure	Mean ± Standard Deviation
Age (years)	15.9 ± 1.8
Height (cm)	160.3 ± 7.4
Weight (kg)	60.2 ± 10.5
BMI (kg/m^2^)	23.4 ± 3.5
IBW%	115.4 ± 17.7
Measure	Median (Range)
Grade	10 (7–14)
Duration of ED (mo.)	10.5 (0.25–48)
Measure	Percentage (N = 20)
Race	White: 12 (60%) Other: 8 (40%)
Ethnicity	Hispanic: 5 (25%) Non-Hispanic: 15 (75%)
Exercise regularly (% yes)	10 (50%)
Previous yoga experience (% yes)	11 (55%)
Diagnosis	OSFED: 15 (75%) AN: 3 (15%) BN: 1 (5%) ARFID: 1 (5%)

*BMI* body mass index, *IBW* ideal body weight, *OSFED* other specified feeding or eating disorder, *AN* anorexia nervosa, *BN* bulimia nervosa, *ARFID* avoidant/restrictive food intake disorder

Five (25%) participants attended all 12 yoga classes and 6 (30%) attended between 7 and 11 classes. Fourteen (70%) participants completed initial follow-up assessments and 11 (55%), final assessments. Using an intention to treat approach, data from the 14 participants who entered the study were included in the final analysis, with baseline scores from the three subjects who discontinued carried across. Both anxiety and depression among participants improved over time. Assessed by the SOM questionnaire, which included Beck Depression Scale, overall depression levels decreased from baseline to study completion [mean 18 (95%CI 15–22) to 10 (95%CI 6–14), adj. *p* = 0.0001]; as did measured clinical state of anorexia nervosa [10 (95% CI 7–12) vs. 6 (95%CI 4–8) , adj. *p* = 0.0004]. Similarly, there was a significant decrease in State anxiety mean scores [47 (95%CI 42–52) to 42 (95%CI 37–47), adj. *p* = 0.0316] after the completion of 7–12 yoga classes.

**Fig. 2:**
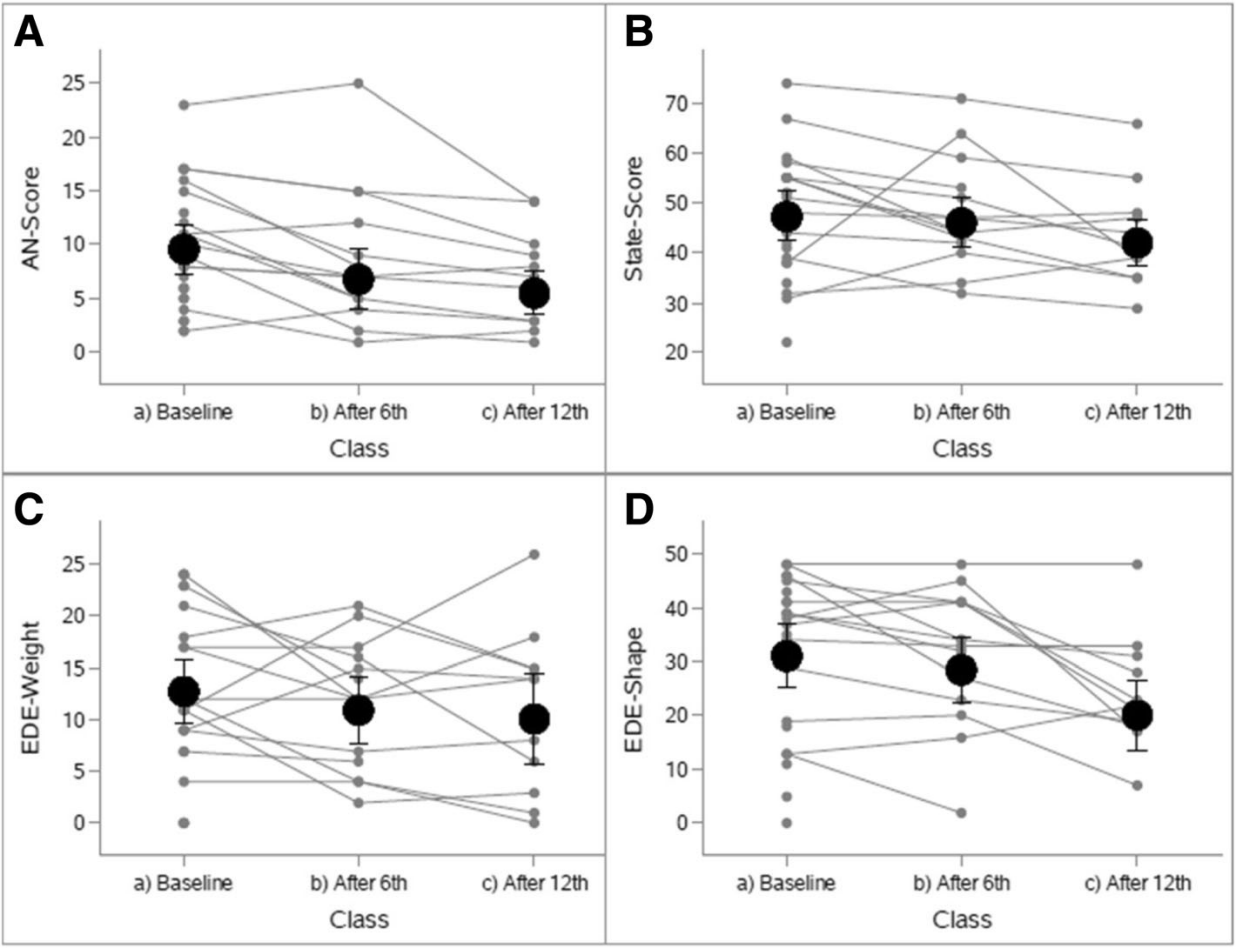
Individual differences in psychological outcome measures at study time points (baseline (*n* = 20), and after 6^th^ (*n* =14) and 12^th^ visits (*n* = 11)

EDE subscales (restraint, eating concern, shape concern, weight concern) were examined individually. The restraint and eating concern subscales did not change during the course of the study while changes in weight concern and shape concern showed a significant reduction. Participants reported significant decreases in weight and shape concerns over the course of the study (Fig. 2), including decreased weight concern [mean 16 (95%CI 12–20) to 12 (95%CI 8–16), adj. *p* =0.0120] and shape concern [31 (95%CI 25–37) to 20 (95%CI 13–27), adj. *p* = 0.0034] comparing the baseline to final (i.e., after 12^th^ class) assessment (Fig. 2).

## Discussion

The Let’s Yoga study aimed to make participation in consistent yoga practice available to a group of adolescents undergoing eating disorder treatment, while examining its effect on their psychological symptoms. Findings from this study suggest that yoga may be an effective adjunct treatment for reducing symptoms of anxiety, depression, and weight and shape concerns in these patients. The decrease in anxiety accompanying lowered weight and shape concerns observed are promising findings that should be further explored as yoga may be especially helpful for those individuals diagnosed with co-existing anxiety, distorted body image and fear of weight gain, a constellation common in the eating disorder population. The current preliminary data hold promise regarding yoga as a beneficial complementary and alternative intervention in the context of the established evidenced based treatments implemented by medical providers, therapists and registered dieticians.

This study aimed to offer physical activity in the form of yoga without the risk of weight loss or medical decompensation. In the design phase of this protocol, our collective experience as a clinical team informed us regarding the desire of this patient group to return to regular physical exercise, sometimes as a form of purging. In the absence of a control group, we cannot rule out the possibility that anxiety and other negative emotions would have improved upon the initiation of any form of physical activity. The subjects’ willingness to participate in any type of research study may also reflect strides towards psychological recovery, accompanied by a receptiveness to any new intervention. Fortunately, the current intervention did not negatively impact BMI or vital signs, suggesting that yoga can provide an opportunity for safe engagement in physical activity. The current findings were not unexpected as the classes attended were reviewed for appropriateness for these patients who also actively attended a clinic that provided consistent medical and nutrition guidance. Importantly, the yoga classes selected were first reviewed by health providers and caretakers to ensure that they were physically and emotionally safe for these patients. This review was necessary as some yoga studios incorporate heat and strenuous physical activity, mirrors, and endorse yoga as a weight loss tool.

The research team did not attend classes with participants as it was the team’s impression that an investigator’s presence had the potential to generate anxiety and disrupt the community connection promoted in the classes. The choice to facilitate classes in a studio versus a clinic setting, as well as allowing patients to select from a variety of classes, was intentional, with the goal to protect participant anonymity, prevent body comparisons between patients and offer schedule flexibility. It was also a concern that participating in a yoga class in the same setting in which patients were receiving medical treatment could be viewed as stressful and might interfere with the relaxation that yoga aims to provide.

Statistically significant decreases in negative emotions were captured on psychological scales among the current participants. These findings were obtained in the context of a brief intervention. Future studies that afford longer term yoga participation would be of interest to determine whether additional and more sustained decreases in anxiety, depression and eating disordered cognitions are observed. The response to recruitment observed and discussion with potential participants suggest interest in the practice of yoga among an adolescent patient group that should be further explored, optimizing factors for an adolescent such as proximity of studio to the clinic, options for transportation, etc.

During the treatment and recovery process, individuals with eating disorders are often confronted with the challenge of refraining from unhealthy behaviors that have become comforting patterns, such as restrictive eating or excessive exercise. In turn, they are asked to participate in distressing actions such as eating foods that cause anxiety, increasing their nutrition intake and refraining from purging. Goals in recovery that can transfer to the practice of yoga include cultivating self-acceptance, respecting personal boundaries, challenging resistance and tolerating discomfort [33]. Although not explored in the current study, as preliminary evidence suggests that yoga practice can support the challenges associated with eating disorder treatment, it would be of interest to explore the benefits of yoga in patients early in their treatment course once they are medically appropriate candidates. This practice may lead to improved adherence to a difficult treatment course. A 2006 study found that two-thirds of residential eating disorders programs surveyed offer yoga [34]. However, due to the extensive variation in experiences of individuals with eating disorders, an assessment of each individual is advised, prior to developing recommendations. A history of excessive exercise, trauma, age and physical abilities should be considered during assessments.

Limitations of the current study merit consideration. The sample size was small, and study attrition resulted in a limited number of participants. However, despite the small numbers studied, statistically significant changes in clinically meaningful outcomes were observed. The study did not include a control group, nor did it offer alternative relaxation methods. However, each participant’s final to baseline measures were compared to assess for changes on validated psychological surveys, which provided pilot data in this patient group after engagement in yoga. Binge eating disorder, recognized as an eating disorder in DSM-5, was not included as it is not treated in the clinic from which patients were recruited. The participants were also limited to cisgender females and did not include males and gender minorities who are at high risk for eating disorders. Findings are, therefore, not generalizable to patients with binge-eating disorders, males and LGBT youth. The reason behind the longer than identified goal of 12 weeks to complete 12 classes include: an already intensive treatment schedule, with medical, nutrition, therapy and psychiatry appointments; medical decompensation and the various levels of care during the course of treatment, including admission to a partial hospital program and residential treatment facility; and worsened adherence to scheduled appointments often as the patient moved closer to recovery from the eating disorder. Future studies should carefully assess for these factors in future research endeavors and initiate countermeasures to keep study subjects engaged. The study was also carried out during the winter months in a northeastern city that experienced a notably severe winter, leading to class cancellations and the inability to travel to classes weekly. Finally, alternative retention strategies should be explored in future studies to improve and optimize adherence to the prescribed yoga classes, including: use of more selective recruitment, with clearer discussion with potential participants about what is required (e.g., consistent weekly attendance at classes in order to test the effects of this intervention), and a design that allows for classes being held on-site at the medical center so that routine medical and mental health appointments could be scheduled on the same day as yoga classes. This latter point is particularly important as young adolescents are dependent on others or public transportation in order to make their weekly appointments.

## Conclusions

In summary, this preliminary study explored an adjunct treatment for adolescents with eating disorders and examined psychological outcomes. Benefits over the 29 weeks of observation were observed with respect to lessened anxiety, depression and weight and body shape concerns. While these initial findings hold promise, the feasibility and efficacy of yoga practice as part of standard outpatient treatment warrants additional investigation. Future studies should include: alternative frequency guidelines, a larger sample size, and a control group (i.e., patients receiving outpatient treatment only) to expand on the current results. An enhanced understanding of this intervention will broaden the treatment options available for this population.
